# The Role of Facial Action Units in Investigating Facial Movements During Speech

**DOI:** 10.3390/electronics14102066

**Published:** 2025-05-20

**Authors:** Aliya A. Newby, Ambika Bhatta, Charles Kirkland, Nicole Arnold, Lara A. Thompson

**Affiliations:** Biomedical Engineering Program, Center for Biomechanical & Rehabilitation Engineering, School of Engineering and Applied Sciences, University of the District of Columbia, 4200 Connecticut Ave. NW, Washington, DC 20008, USA

**Keywords:** speech, facial action units, apraxia of speech, speech sound disorders

## Abstract

Investigating how facial movements can be used to characterize and quantify speech is important, in particular, to aid those suffering from motor control speech disorders. Here, we sought to investigate how facial action units (AUs), previously used to classify human expressions and emotion, could be used to quantify and understand unimpaired human speech. Fourteen (14) adult participants (30.1 ± 7.9 years old), fluent in English, with no speech impairments, were examined. Within each data collection session, 6 video trials per participant per phoneme were acquired (i.e., 102 trials total/phoneme). The participants were asked to vocalize the vowels /æ/, /ɛ/, /ɪ/, /ɒ/, and /ʊ/; the consonants /b/, /n/, /m/, /p/, /h/, /w/, and /d/; and the diphthongs /eI/, /ʌɪ/, /i/, /a:/, and /u:/. Through the use of Python Py-Feat, our analysis displayed the AU contributions for each phoneme. The important implication of our methodological findings is that AUs could be used to quantify speech in populations with no speech disability; this has the potential to be broadened toward providing feedback and characterization of speech changes and improvements in impaired populations. This would be of interest to persons with speech disorders, speech language pathologists, engineers, and physicians.

## 1. Introduction

In everyday life, the ability to speak and be understood is something many of us take for granted. However, motor control impairments tied to speech can be debilitating. Disorders wherein the muscles used for speaking are dysfunctional due to neurological damage lead to difficulties with articulation, pronunciation, and clarity of speech [1]. Such impairments are commonly categorized as either dysarthria (muscle weakness) or apraxia of speech (difficulty planning and coordinating muscle movements for speech production) [2]. In adults, these disorders can arise from a variety of causes (e.g., stroke, brain tumors, multiple sclerosis, Parkinson’s disease, amyotrophic lateral sclerosis (ALS), cerebral palsy, head trauma, or neurodegenerative diseases) [3]. In children, speech motor control impairments can arise from conditions such as childhood apraxia of speech (CAS) [4]. Childhood apraxia of speech and developmental dysarthria may co-occur with organic syndromes, such as Down Syndrome, Williams–Beuren Syndrome, and Cri-du-Chat Syndrome that also present auxiliary difficulties [5,6], or could occur independent of these.

Currently, how speech language pathologists (SLPs) assess speech disorders is through subjective yet comprehensive assessment, case history of the subject and their family, performing an oral mechanism examination, hearing screening, and speech sound assessment. During the evaluation of speech, the accuracy of speech production, speech sound errors, and error patterns are observed by the SLP [7]. Intelligibility assessments are administered to help pathologists determine the severity of speech difficulty using subjective rating scales [8]. Speech is also evaluated with the use of objective measures and indices, such as the percentage of consonants correct and whole-word matches [9]. However, this approach results in several limitations: metrics such as the type and frequency of speech sound errors are usually quantified using rating scales; the speaker’s articulation approach and linguistic factors are challenging to interpret by the SLP; the listener’s (SLP’s) familiarity with the speaker’s (patient’s) speech pattern may vary; communication cues for the person being assessed may be misinterpreted; and the presence of background noise may affect the results of an intelligibility assessment [10]. Stimulability tests are administered to determine how a child articulates sounds in various contexts [11]. However, a major limiting factor includes variations in the way the test is administered (by different clinicians), leading to inconsistencies in the results. Speech perception tests allow the SLP/clinician to determine the patient’s ability to perceive the difference between the standard production. Limitations of this method include speech presentations that may not reflect how the subject speaks in everyday situations and the difficulty of use in younger children (i.e., misleading results due to the child’s misunderstanding and/or misinterpretation of the procedural requests).

For all of the above, a qualitative judgment, as opposed to a quantitative indication, is made by the SLP on how the speech sound disorder impacts functional communication. While the above clinical tests administered by the SLPs have proven to be valuable, there are several other limitations to consider [12]. These limitations include subjective bias and interpretation of the SLP, cultural and linguistic biases, lack of contextual relevance, and an overreliance on and confidence in traditional assessments, thus leading to potential for misdiagnosis [13]. Further, assessments must be tailored for children who are too young to be evaluated using the above, adults or children who are either reluctant or unable, and adults and children with less intelligible speech (i.e., more severe speech deficits) [14]. Furthermore, it is known that children have relatively smaller attention spans while undergoing instruction and training [15]. Attention is considered very critical in cognitive learning [16]. Some individuals with speech disorders may also have other exacerbating conditions and may therefore have difficulty either performing or comprehending traditionally used, standardized tests; thus, they would be unable to provide the requested responses. Alternative tests that are objective, as well as applicable across a wider range of individuals with speech deficits, require further inspection and development [17].

The studies below use vastly different approaches, and different goals than our study, which utilized facial action units (AUs) (linked to facial muscle groups), to examine the production of speech (phonemes). An audio–visual speech recognition method previously utilized side-face images of the lips, which are captured while the audio is recorded using a microphone. Lip contour geometric features (LCGFs), used for discriminating phonemes, and lip movement velocity features (LMVFs), used for detecting voice activity, were used to extract both audio and visual features. These features were used to assess speech using Hidden Markov Models (HMMs) [18]. Another study designed and compared two HMM classifiers for four emotions which have varying effects on the properties of different phonemes. The emotional states during speech were classified using phoneme-level modeling [19]. Phonetic feature-based prediction models were used to predict and understand variations in the pronunciation of phonemes. This allowed for better modeling of spontaneous speech by focusing on individual articulatory features [20]. Another approach focused on identifying the relationship between speech sounds and facial movements using a fine-grained statistical correlation analysis between various phonemes and facial anthropometric measurements (AMs) [21]. Another alternative to phoneme analysis using facial movements is the viseme model. Viseme maps are particularly useful in supporting accurate visual feature extraction for the visual representation of speech [22,23].

On the other hand, techniques like electropalatography (EPG) or palatography involve embedding an electrode to the palate to monitor tongue movements. Though non-invasive and shown to be effective in children and adults [24], these have not been proven to be reliable for vowels and glides where vocal tract constrictions have greater contribution [25]. Also, EPG therapy may not be as advantageous, especially for younger children who have not shown a resistance to traditional methods. It is important to note that many facial expressions are innate. In other words, facial expressions do not vary between a person blind from birth and an individual with progressive loss of sight [26,27]. Intuitively, the perception of speech, especially in a noisy environment, is better due to visual details [28]. Furthermore, the speech voice features independent of vocal tract characteristics will need other reliable estimators to develop speech–face mapping [29,30]. This is shown through some unique facial traits identifiers with acoustic models. This establishes a substantial use of facial expression for improved learning ability by visual and sound cues without discarding that Visemes are a subset of phonemes, and work related to Visemes will further enhance the presented investigation [31]. Thus, these findings establish the important impact of the presented AU acoustic-based assessment feedback approach [32–34].

In our research, we explored the potential for objective quantification of speech sounds involving measurement of facial muscle groups’ movements during speech via facial action units (AUs). We hypothesized that phonemes could be assessed and quantified via AU mapping. The facial action coding system (FACS) previously was developed primarily for the analysis and distinction of facial expressions toward human emotions [35]; however, here, we aimed to utilize AUs to quantify facial movements in relation to speech. Automated facial analysis includes facial detection and tracking, feature extraction, and classification, where the facial regions categorized by action units are detected from image frames extracted from videos. This approach has potential to allow for an objective, customized assessment for individual speech based on personal features. Our goal was to determine AU responses to define a baseline for normal speech (for unimpaired participants). This information can later be contrasted with impaired speech and used to provide feedback for speech training.

## 2. Materials and Methods

All study activities were conducted within the Center for Biomechanical and Rehabilitation Engineering (CBRE) laboratory at the University of the District of Columbia (Washington, DC, USA). The protocol was approved by the Institutional Review Board (2248324–1), and all participants gave their informed consent prior to participating in the study.

### 2.1. Participants

Participants learned about the study through flyers posted around the university and word of mouth. The population targeted were adults (18–45 years old) who were fluent, American English speakers without speech impairments; here, we did not examine non-English-speaking participants. Participants provided their informed consent prior to taking part in the research study. Fourteen participants were enrolled in the study and their demographics are shown in Table 1. After participants gave their informed consent, their data collection session was scheduled.

### 2.2. Data Collection

**Figure 1.**
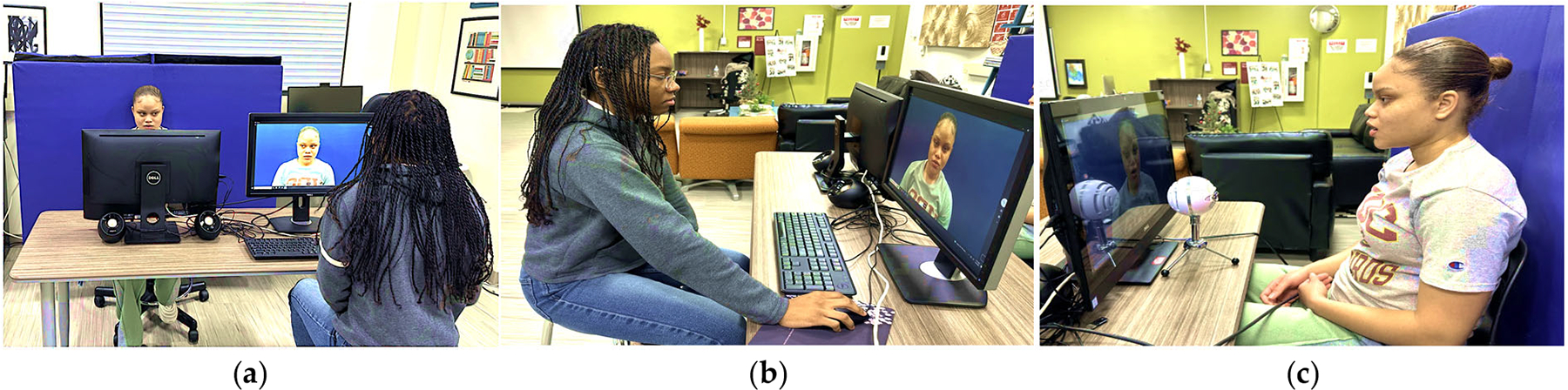
Example of AU data collection session with a participant: (**a**) experimental setup for data collection (desktop computer with researcher; participant facing camera and microphone); (**b**) researcher controlling camera application and recording speech audio; (**c**) participant vocalizing during the trial.

The experimental setup involved the use of the following equipment: a desktop computer, connected to an external microphone and speakers, as well as an additional desktop computer for the researcher to control the camera application while recording the participants as they articulate the sounds. Prior to the session, the research assistants ensured that the experimental setup was fully functional. The setup process involved turning on the desktop computers, recording a test video to confirm the functionality of the desktop’s camera application, and ensuring the video quality was sufficient. The desktop’s inbuilt camera was used to facilitate the position and circumvent the need of installing drivers for portable cameras in different systems. The objective was to develop a portable audio- and video-capturing system. Figure 1 shows the experimental setup used for recording the data.

A solid-colored background was placed behind the participant’s chair and adjusted to ensure it was the only visible background in the videos (Figure 1). The participant’s chair was positioned so that only the participant’s head, neck, and upper shoulders were visible in the recording on the computer. Priority was given to the visibility of the participant’s face; therefore, participants were asked to remove hats, jewelry, glasses, bulky clothing, or any other items that could interfere with facial visibility. The distance between the chair and the background was adjusted to accommodate variations in participant height. The recording desktop screen was controlled by the desktop computer manned by the researcher. To minimize background noise, additional movements in the lab were restricted during the recording sessions. The desktop’s camera and microphone and an additional microphone (for improving the sound quality of the participants’ voice) were used to record each participant while vocalizing phonemes (vowels, phonemes, and diphthongs). The recorded video format is MP4, with a bit depth of 8, frame rate of 29.8 frames per second, and resolution of 1920 × 1080. The audio format is MP3 with 2 channels, 1024 samples/frame, and a sampling rate of 44,100 Hz.

During the session, the researcher sat at the opposite side of the table, facing a desktop that allowed full control of the recording process. This setup ensured that participants only needed to speak when cued. Participants were prompted to look directly into the desktop’s camera lens for the duration of the recording. They were informed that a beep would indicate the start of the recording, after which the participant would produce the designated phoneme sound six times. Before recordings began, the experimenter provided instructions and asked the participant if any steps were unclear. With the test setup in place, video recording was ready to proceed.

Once the video recording was started, the participant proceeded with vocalizing each phoneme. The following phonemes were repeated 6 times per video recording, with breaks between recordings: /æ/, /ɛ/, /ɪ/, /ɑ/, /ɒ/, /b/, /n/, /m/, /p/, /h/, /w/, /d/, /eɪ/, /ʌɪ/, /i/, /a:/, and /u:/. The participant produced the sound six times with an estimated one-second interval between repetitions. After the recording for each phoneme ended, the participant was given a brief break before proceeding with the next recording.

At the end of the session, the participants’ videos (17 videos per participant, with 6 repetitions per phoneme) were stored in a separate folder on the computer. Participants were informed that their speech analysis results would be provided near the end of the study and were encouraged to reach out with any questions. In the presented data collection, ensuring sufficient natural or white light exposure was strictly considered and guaranteeing maximum coverage of the head shot in the field of view (FOV) was emphasized. For acoustic sound recording, any ambient noise was restricted by enclosing the recording room. A Logitech Snowball Microphone was used for its better directive features. However, no detailed study on the negative impact of reverberation and background noise has been presented here. Some apparent noises were edited out using Audacity (https://www.audacityteam.org/) and other speech processing software.

### 2.3. Data Processing

**Figure 2.**
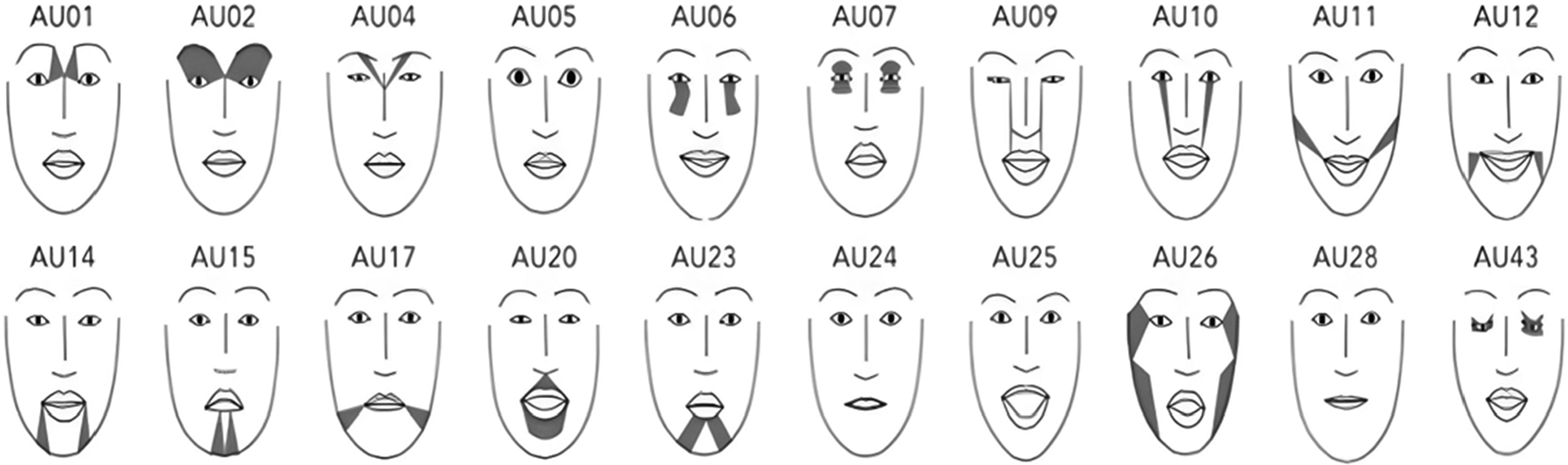
Mapping and numbering of the facial action units (AUs) [36].

Once the videos were preprocessed to ensure each trial was separated, the Py-Feat: Python Facial Expression Analysis Toolbox (Python, software version 3.9) was used to determine action units (AUs), or facial muscle groups, used for each phoneme and word presentation. AUs are numbered muscle groups of the face, used to produce particular facial expressions—in this case, facial movements (Figure 2). A total of 20 AUs were generated with 11 specifically involved in speech production (e.g., facial muscle groups surrounding the mouth, cheeks, and jaws) (Table 2).

The videos were separated into individual trials and processed as separate videos; each video trial was converted into frames. All video preparations were carried out using the scripting language, Python. This facilitated time-intensive steps of converting recorded videos into a programmable format. The platform for analyzing and processing the vocalization videos was IDLE (integrated development environment for Python), using the PyFeat Facial Expression Recognition (FER) module [36]. The outputs generated from processing the videos are frames saved as jpeg. files with the corresponding contour maps for each frame. This allowed for visualization of the AUs representing the activated facial muscles. The summary of AUs and averages across frames were saved as csv. (comma-separated value) files for each of the recorded phonemes.

The results were pooled across participants for each phoneme to generate a boxplot for each AU. Within the boxplots, median, upper quartile range, lower quartile range, and outliers were determined for each phoneme as a function of AU. Boxplots were created to show the distribution in mean AU amplitude across all trials for each sound. Action units with the least variation in mean AU amplitude and a higher median indicated the most activated facial muscles specific to these sound types across all participants. A benchmark was developed to establish the range of mean AU amplitude for the AUs representing facial muscles involved in unimpaired speech production. This range varies depending on the phoneme.

## 3. Results

**Figure 3.**
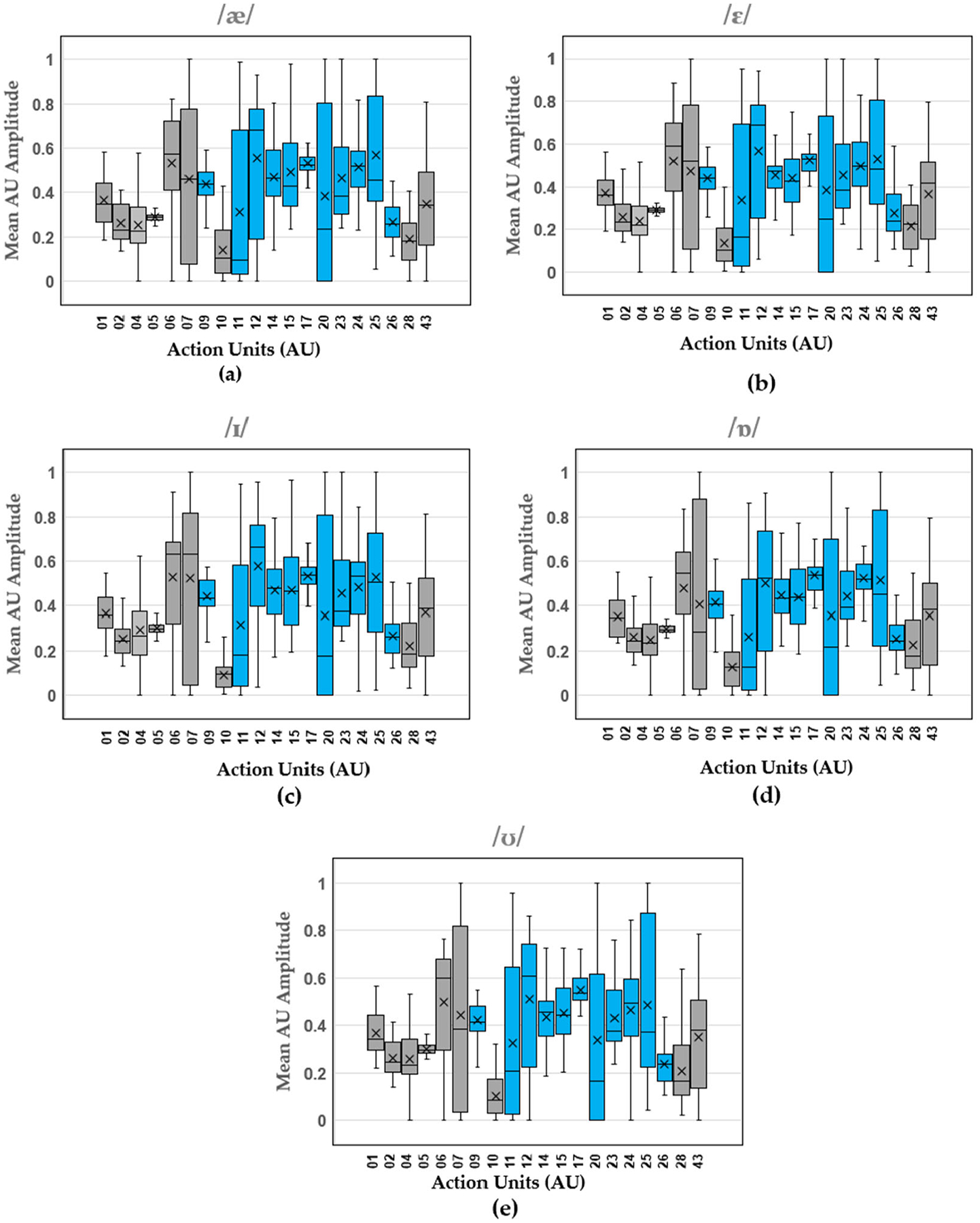
Vowel AU amplitude boxplots (median, upper and lower interquartile range (IQR), and standard deviations pooled across participants) for (**a**) /æ/, (**b**) /ε/, (**c**) /ɪ/, (**d**) /ɒ/, and (**e**) /ʊ/). Blue boxes indicate AUs specifically related to speech, while gray boxes represent AUs not associated with speech.

**Figure 4.**
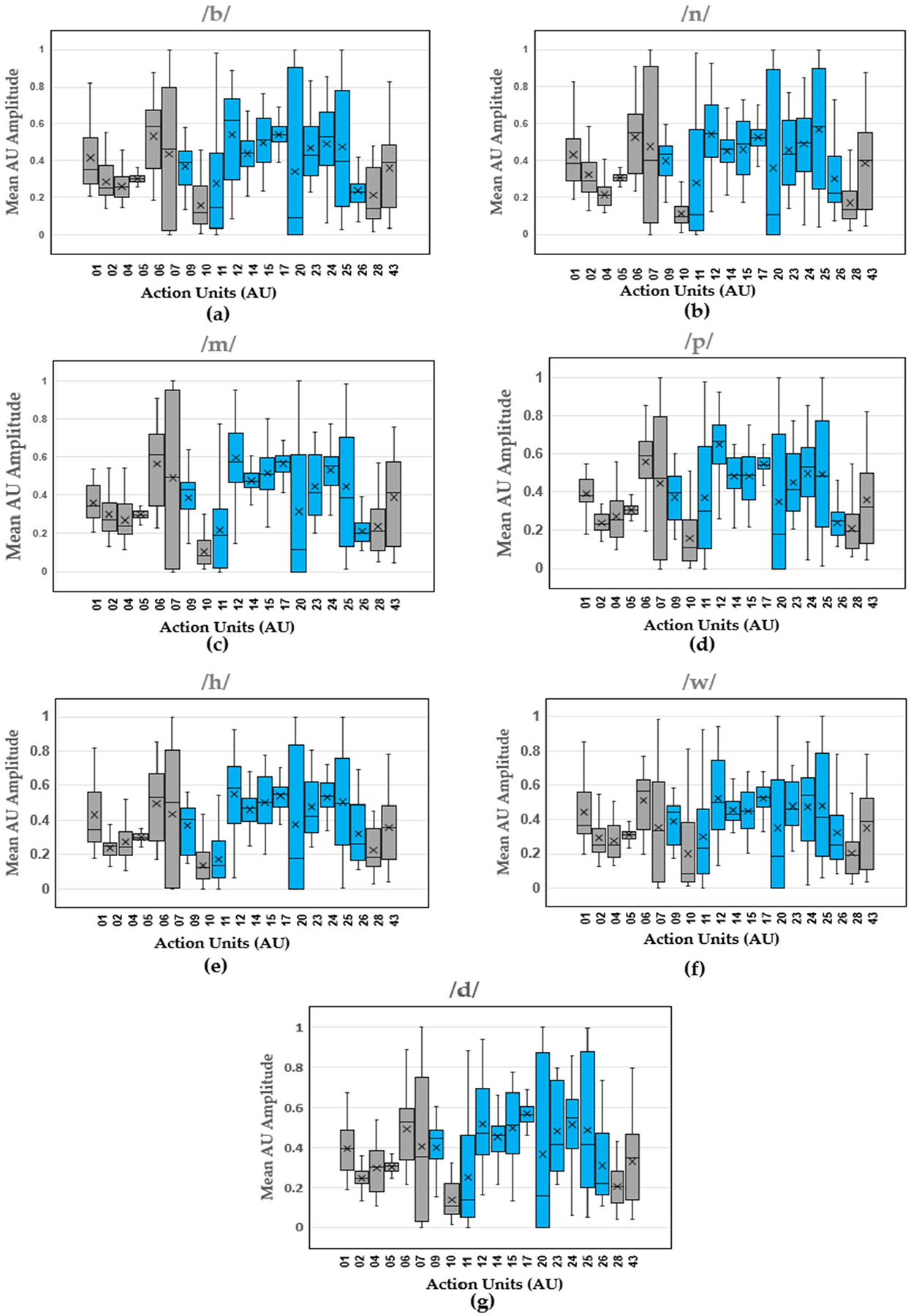
Consonant AU amplitude boxplots (median, upper and lower interquartile range (IQR), and standard deviations pooled across participants) for (**a**) /b/, (**b**) /n/, (**c**) /m/, (**d**) /p/, (**e**) /h/, (**f**) /w/, and (**g**) /d/. Blue boxes indicate AUs specifically related to speech, while gray boxes represent AUs not associated with speech.

**Figure 5.**
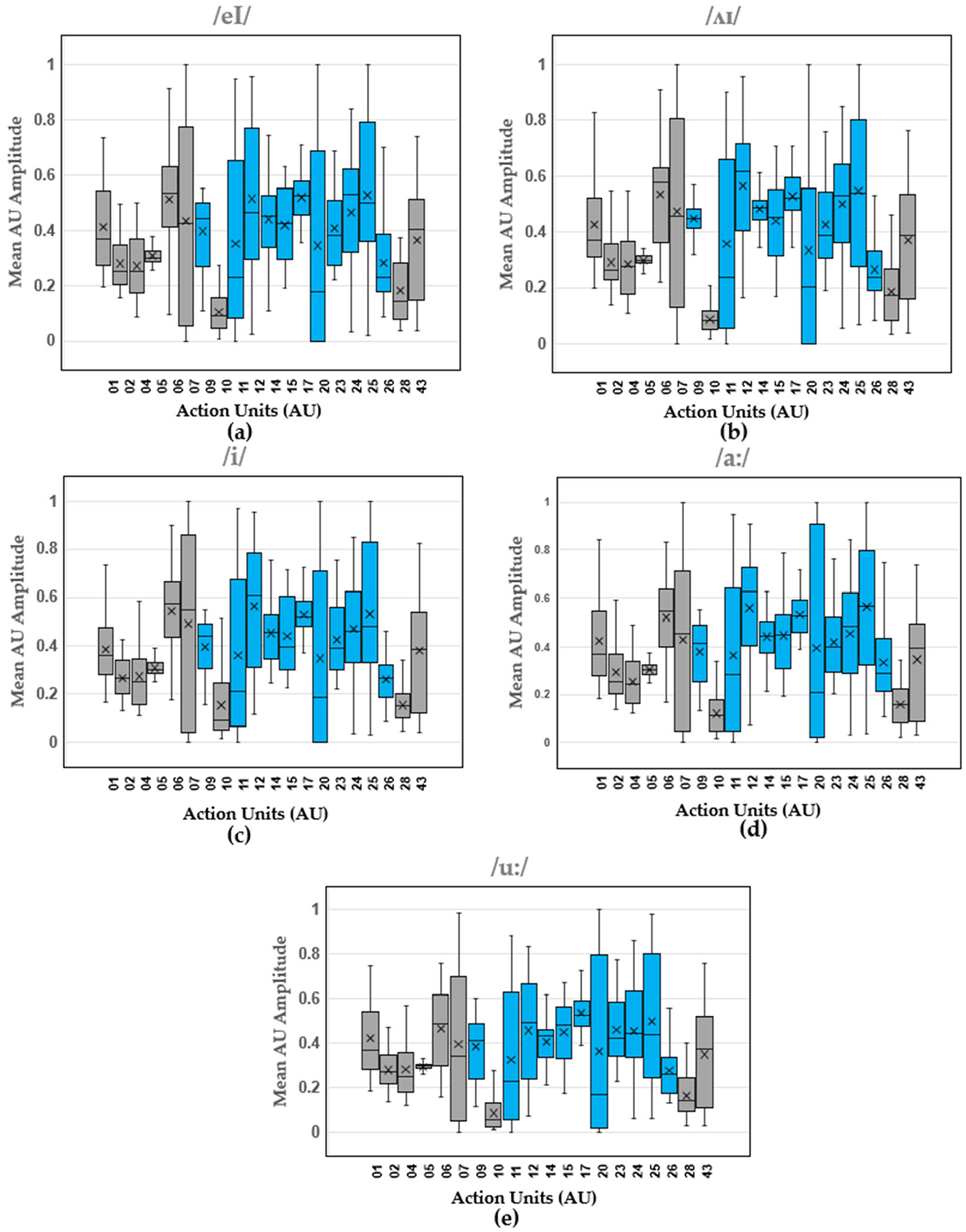
Diphthong AU amplitude boxplots (median, upper and lower interquartile range (IQR), and standard deviations pooled across participants) for (**a**) /eI/, (**b**) /ʌɪ/, (**c**) /i/, (**d**) /a:/, and (**e**) /u:/. Blue boxes indicate AUs specifically related to speech, while gray boxes represent AUs not associated with speech.

Boxplots show the distribution of mean AU amplitude for the different action units across all the trials produced by all the participants. The results for the vowels (Figure 3), consonants (Figure 4), and diphthongs (Figure 5) are shown.

Table 3 shows the median values of AU amplitude for each phoneme.

## 4. Discussion

Within the results, movement of the articulators, as reflected in the activity of specific facial muscle groups, was observed. More specifically, our study analyzed the median amplitude and distribution of action units (AUs) associated with facial muscle groups during the articulation of various speech sounds, including vowels, phonemes, and diphthongs. The articulators (tongue, lips, and jaw) play a critical role in speech production and are primarily controlled by muscles in the face. The findings are presented through boxplots displaying the median for the AU amplitude values of each articulated sound, providing a visual representation of the variation in AU amplitudes for different sounds and allowing for an objective assessment of the muscle groups most engaged during speech production. The median values across the different AUs indicate the central tendency of muscle activation in speech articulation across participants. Table 3 shows the range of AU activation, where the most activated AUs are represented by the darkest shades of blue and the least activated AUs are represented by the lightest shades of blue.

The analysis of vowel sounds reveals distinct activation patterns across the 11 facial AUs contributing to speech production. Notably, the AU12 (Nasolabial deepener) showed the highest activation in vowels /æ/, /ɛ/, and /ʊ/, with the exception of AU17 (Lip corner depressor) which showed the highest activation for vowel /ɪ/, and vowel /ɒ/ shows the highest activations of muscle groups represented by AUs 09 (Nose wrinkler), 12 (Nasolabial deepener), 14 (Lip corner puller), 15 (Dimpler), 17 (Lip corner depressor), 23 (Lip stretcher), 24 (Lip tightener), and 25 (Lip pressor). It was more challenging to isolate the most activated action unit based on the median values for consonants displayed in Table 3. The most activated AU for consonants /b/ and /p/ was AU12 (Nasolabial deepener). The remaining consonants, /n/, /m/, /h/, /w/, and /d/ showed the highest activation in muscle groups represented by AUs 09 (Nose wrinkler), 12 (Nasolabial deepener), 14 (Lip corner puller), 15 (Dimpler), 17 (Lip corner depressor), 23 (Lip stretcher), 24 (Lip tightener), and 25 (Lip pressor). Given that these consonants are not characterized by a single most activated AU, it is then recommended to pay close attention to these muscle groups during the articulation of these sounds. It is also important to note that the boxplots shown for AUs of particular interest in speech are mostly skewed, indicating the outliers and scope for increasing the sample size when developing AI and machine learning models for feedback interfaces. The latter has not been discussed in this paper and is mentioned here for its potential application [38]. The presented work is an exposition for developing AU-based modeling of normal speech of sounds and words assuming the sample size, gender, age, and other variability are secondary as opposed to primary factors in facial muscle activation. Hence, the accuracy and confidence of generalizing the model has not been stated and will be of greater importance in studies following the presented work.

AU12 (Nasolabial deepener) also showed the highest activation during the articulation of diphthongs /ʌɪ/, /i/, and /a:/. On the other hand, the AUs with the highest activation were 09, 12, 14, 15, 17, 24, and 25 for diphthong /eI/ and AUs 09, 12, 14, 15, 17, 23, 24, and 25. Identifying the AUs that are most activated during unimpaired speech could potentially help speech therapists focus on the muscle groups represented by these AUs, allowing them to customize their treatment approaches to meet individual patient needs.

While preliminary, these results highlight aspects that could potentially be used to develop robust measures for improving and training individuals with impaired speech. The observed differences in AU activation patterns across different sound types can inform speech therapy and rehabilitation techniques for individuals with speech impairments. These insights can also be applied to the development of facial recognition and speech synthesis technologies.

A similar approach was taken in a previous study for the application of facial action units in the analysis and prediction of stuttering speech [39]. Facial AUs were then extracted from video recordings of participants and calculated from participants’ faces using a model trained with various datasets. The AUs were then categorized based on the upper and lower facial regions. The facial movements represented by the different AUs were recorded and processed as inputs for a deep learning model used to distinguish between fluent and stuttered speech. A distinction was made between the AUs that contributed to a higher likelihood of stuttering and those that contributed the least or not at all to the prediction of stuttering. It was shown that facial AUs contributed significantly to distinguishing between fluent and stuttered speech while also providing insights into the non-verbal cues related to stuttering. Limitations of this study included variations in facial structures and expressions among individuals, which may have made it challenging to generalize the results across different individuals; the limited resolution of the recordings used to capture changes in facial muscle movements; the differences in the study setup, which could cause variations in results when applied to real-time stuttering; and the possibility that some AUs were miscategorized or excluded from the model, potentially leading to misunderstandings of the contributions of AUs in this study.

Future research could further categorize the data for a more detailed analysis of individual variations in speech production, as well as the impact of external factors such as speech rate and emotional expression. Our study provides insight into how facial muscle activation varies across speech sounds, emphasizing the role of the articulators in speech production. This serves as a baseline toward the application of this method to characterize the speech of individuals (children and adults) with speech and language disorders. We aim to increase the sample size of participants and the time allocated for data collection in future studies.

## Figures and Tables

**Table 1.** Overview of participant demographics.

Participant	Gender	Age	Ethnicity	Traumatic Brain/Stroke Injury	Cognitive Impairments	Development Delays	Learning Disability and/or Speech Impairment
S1	F	26	Black/African American	No	No	No	No
S2	F	46	Black/African American	No	No	No	No
S3	F	38	Black/African American	No	No	No	No
S4	M	26	Asian/Pacific Islander	No	No	No	No
S5	M	21	Black/African American	No	No	No	No
S6	F	41	Asian/Pacific Islander	No	No	No	No
S7	M	25	Black/African American	No	No	No	No
S8	F	29	Black/African American	No	No	No	No
S9	M	25	Black/African American	No	No	No	ADHD, Graphomotor Disorder
S10	M	23	Black/African American	No	No	No	No
S11	F	26	Black/African American	No	No	No	No
S12	M	28	Hispanic/Latino	No	No	No	No
S13	F	43	Black/African American	No	No	No	No
S14	F	24	Black/African American	No	No	No	No

**Table 2.** Action units (AUs) specifically used for speech production [37].

Action Units	Muscle Groups	Description of Muscle Group Function
AU09	Levator Labii Superioris Alaeque Nasi	Nose wrinkler
AU11	Zygomaticus Minor	Nasolabial deepener
AU12	Zygomaticus Major	Lip corner puller
AU14	Buccinator	Dimpler
AU15	Depressor Anguli Oris	Lip corner depressor
AU17	Mentalis	Chin raiser
AU20	Risorius, Platysma	Lip stretcher
AU23	Orbicularis Oris	Lip tightener
AU24	Orbicularis Oris	Lip pressor
AU25	Depressor Labii Inferioris	Lip part
AU26	Masseter, Temporalis, Medial Pterygoid	Jaw drop [36,37]

**Table 3.** Median values of AU amplitude for facial action units specific to articulated sounds; red text indicates those AUs specific to speech.

	Vowels					Consonants							Diphthongs				
Range of AUs	/æ/	/ε/	/ɪ/	/ɒ/	/ʊ/	/b/	/n/	/m/	/p/	/h/	/w/	/d/	/eI/	/ʌɪ/	/i/	/a:/	/u:/
AU01	0.375	0.371	0.368	0.35	0.35	0.35	0.38	0.35	0.39	0.34	0.37	0.42	0.37	0.37	0.37	0.37	0.37
AU02	0.250	0.239	0.25	0.26	0.25	0.25	0.29	0.27	0.26	0.26	0.25	0.26	0.25	0.26	0.27	0.25	0.27
AU04	0.24	0.264	0.27	0.25	0.23	0.26	0.24	0.24	0.25	0.25	0.25	0.3	0.25	0.28	0.25	0.24	0.25
AU05	0.3	0.297	0.63	0.30	0.30	0.30	0.31	0.31	0.3	0.30	0.31	0.31	0.30	0.30	0.30	0.31	0.30
AU06	0.59	0.592	0.63	0.54	0.6	0.59	0.55	0.61	0.59	0.53	0.56	0.53	0.53	0.58	0.57	0.55	0.49
AU07	0.45	0.52	0.42	0.28	0.38	0.46	0.4	0.5	0.47	0.5	0.34	0.35	0.43	0.45	0.55	0.45	0.34
AU09	0.425	0.425	0.12	0.40	0.4	0.39	0.43	0.43	0.4	0.4	0.44	0.45	0.44	0.43	0.44	0.41	0.41
AU10	0.175	0.13	0.66	0.14	0.09	0.12	0.11	0.11	0.13	0.13	0.08	0.13	0.1	0.11	0.1	0.13	0.1
AU11	0.075	0.165	0.5	0.125	0.21	0.15	0.1	0.21	0.3	0.21	0.24	0.13	0.23	0.24	0.21	0.28	0.23
AU12	0.695	0.691	0.47	0.52	0.61	0.62	0.54	0.58	0.66	0.58	0.5	0.47	0.46	0.62	0.61	0.63	0.5
AU14	0.475	0.477	0.54	0.43	0.46	0.44	0.46	0.48	0.46	0.47	0.43	0.46	0.45	0.48	0.46	0.44	0.43
AU15	0.45	0.45	0.18	0.45	0.45	0.51	0.49	0.51	0.46	0.5	0.45	0.51	0.42	0.45	0.39	0.45	0.48
AU17	0.55	0.53	0.66	0.54	0.54	0.54	0.52	0.58	0.54	0.55	0.53	0.55	0.53	0.52	0.52	0.53	0.53
AU20	0.21	0.25	0.49	0.22	0.17	0.09	0.11	0.12	0.18	0.18	0.18	0.16	0.18	0.2	0.19	0.21	0.17
AU23	0.39	0.38	0.46	0.41	0.39	0.43	0.43	0.41	0.41	0.42	0.46	0.42	0.38	0.39	0.39	0.4	0.42
AU24	0.5	0.49	0.53	0.51	0.49	0.53	0.49	0.56	0.53	0.53	0.54	0.55	0.53	0.53	0.46	0.48	0.44
AU25	0.475	0.48	0.51	0.45	0.37	0.4	0.58	0.39	0.48	0.5	0.42	0.41	0.5	0.54	0.48	0.56	0.44
AU26	0.275	0.243	0.27	0.25	0.25	0.25	0.22	0.23	0.27	0.26	0.25	0.22	0.26	0.24	0.29	0.29	0.27
AU28	0.19	0.24	0.19	0.18	0.16	0.14	0.13	0.21	0.2	0.19	0.2	0.2	0.14	0.17	0.16	0.13	0.14
AU43	0.18	0.417	0.39	0.38	0.38	0.39	0.4	0.41	0.32	0.36	0.39	0.35	0.4	0.39	0.39	0.4	0.38
Legend for Median Ranges																	
0–0.2	0.21–0.39	0.4–0.59	0.6–0.79	0.8–1.0													

For the table displayed above, the red text indicates the AUs that are specifically related to speech while the black and white texts indicate the AUs that are not associated with speech production.

## Data Availability

The raw data supporting the conclusions of this article will be made available by the authors on request.
